# Astroglial and Microglial Purinergic P2X7 Receptor as a Major Contributor to Neuroinflammation during the Course of Multiple Sclerosis

**DOI:** 10.3390/ijms22168404

**Published:** 2021-08-05

**Authors:** Marta Sidoryk-Węgrzynowicz, Lidia Strużyńska

**Affiliations:** Laboratory of Pathoneurochemistry, Department of Neurochemistry, Mossakowski Medical Research Institute, 02-106 Warsaw, Poland

**Keywords:** purinergic receptors, neuroinflammation, autoimmune disease, microglia, astroglia

## Abstract

Multiple sclerosis (MS) is an autoimmune inflammatory disease of the central nervous system that leads to the progressive disability of patients. A characteristic feature of the disease is the presence of focal demyelinating lesions accompanied by an inflammatory reaction. Interactions between autoreactive immune cells and glia cells are considered as a central mechanism underlying the pathology of MS. A glia-mediated inflammatory reaction followed by overproduction of free radicals and generation of glutamate-induced excitotoxicity promotes oligodendrocyte injury, contributing to demyelination and subsequent neurodegeneration. Activation of purinergic signaling, in particular P2X7 receptor-mediated signaling, in astrocytes and microglia is an important causative factor in these pathological processes. This review discusses the role of astroglial and microglial cells, and in particular glial P2X7 receptors, in inducing MS-related neuroinflammatory events, highlighting the importance of P2X7R-mediated molecular pathways in MS pathology and identifying these receptors as a potential therapeutic target.

## 1. Introduction

Multiple sclerosis (MS) is a chronic immune-mediated inflammatory disease of the central nervous system (CNS) with unknown etiology that mainly affects young adults, leading to the progressive physical disability and mental stress of patients. This multifactorial disease is characterized by infiltration of activated peripheral immune cells into the brain and spinal cord with subsequent immune-mediated demyelination and neurodegeneration accompanied by inflammation. The presence of focal demyelinating lesions in the white and grey matter of brain and spinal cord of MS patients, connected with reactive micro- and astrogliosis, is a main diagnostic hallmark of the disease. The pathological basis of these lesions is a selective and primary disruption of myelin sheaths with subsequent axonal degeneration and loss of neurons. Development of lesions in cerebral cortex characterizes the evolution from an early relapsing/remitting phase (RR) into a secondary progressive (SP) phase of the disease [1].

The clinical symptoms of the disease, such as imbalance, muscle weakness, motor dyscoordination, pain, paralysis, cognitive impairment, and depression [2], are correlated with the injured anatomical region of the CNS. The MS etiology is still unknown, although genetic, hormonal, and environmental factors are indicated as significantly influencing the development of the disease. A recent study using single-nucleus RNA sequencing demonstrated region-specific transcriptomic alternations associated with selective damage of neurons in cortical layers associated with the upregulation of stress pathway genes and long non-coding RNAs. This study revealed the vulnerability of oligodendrocytes, reactive astrocytes, and activated microglia which appear most frequently in the areas surrounding MS lesions [3].

Activated glial cells, both microglia and astroglia, substantially contribute to MS pathogenesis and progression by driving and accelerating inflammatory reaction, generating free radicals and releasing cytotoxic excitatory amino acids. All these factors contribute to oligodendrocyte injury, myelin damage, and axonal degeneration, finally leading to the death of neurons. Extracellular ATP (eATP) and plethora of its receptors are of importance in neuron–astrocyte–microglia intercommunication in pathological mechanisms occurring in MS. Recent studies demonstrated that glial cells expressing P2X7 receptors (P2X7Rs) are involved in eATP-dependent signaling involved in the crucial pathological mechanisms at early and progressive stages of MS and experimental autoimmune encephalomyelitis (EAE) [4].

This review focuses on the role of microglial and astroglial cells in MS pathology based on clinical and experimental data, and discusses the involvement of P2X7 purinergic receptors into glia-mediated neuroinflammation.

## 2. Multiple Sclerosis and Neuroinflammation

### 2.1. Neuroinflammation during the Course of MS

Focal demyelinated lesions are present in the white matter, as well as in the grey matter of cortex, the basal ganglia, brain stem, and spinal cord [5]. The inflammatory reaction is initiated around post-capillary venoles and veins, spreading further into the surrounding normal-appearing white and grey matter [6]. Perivascular inflammatory infiltrates are composed of T cells, B cells, and immunoglobulin-positive plasma cells, whereas active demyelinating plaques consists mainly of macrophages and activated microglia. CD3+, CD4+, and CD8+ T cells outnumber CD20-positive B cells and plasma cells [7,8], although a subset of inflammatory immune cells differs in composition depending on the brain region (perivascular area vs. parenchymal space) and the stage of the disease (initial vs. advanced) [5]. Activated autoreactive CD4+ cells release pro-inflammatory mediators, such as IFN-γ and/or IL-17, thus driving inflammation. Monocytes recruited from the blood in response to chemokine signaling also constitute a significant component of myelin lesions. Their concentration in lesions has been shown to be positively correlated with the severity of neurological deficits during EAE, whereas depletion of monocytes delays the onset and severity of the disease [9,10]. Activated by cytokines/chemokines, including GM-CSF, INF-γ, and TNF-α, monocytes are transformed into a pro-inflammatory M1 phenotype, expressing MHC II antigens and producing pro-inflammatory factors, proteases, as well as reactive oxygen (ROS) and nitrogen (NRS) species [11]. Thus, migration of immune cells via the blood–brain barrier (BBB), in order to colonize the CNS is a critical step of the disease, contributes to the development of inflammatory cascade. During the course of the disease, in a progressive phase, massive inflammation is observed and mediated by CD4+, CD8+ cells, B cells, and monocytes, and amplified by activated glial cells in response to the presence of disturbed myelin and damaged tissues [12]. The spreading inflammatory process is characterized by upregulation of cytokines, chemokines, and adhesion molecules of both a pro- and anti-inflammatory nature [13]. This situation is aggravated by dysfunction of regulatory T lymphocytes (Tregs) that physiologically suppress autoimmune processes but are impaired in MS patients [14]. 

MS is considered as an inflammatory disease with a neurodegenerative component, in which an autoimmune inflammatory reaction is accompanied by degeneration of the demyelinated nerve fibers. However, the issue has not been resolved within this sequence of events [15]. Oxidative stress [16] and glutamate-induced excitotoxicity [17] are the main mechanisms, contributing significantly to neurodegeneration in MS pathology, in which glial cells are strongly involved. 

### 2.2. Microglia as Contributors to MS-Related Neuroinflammation 

Microglia are resident immune competent cells of the CNS, guarding the homeostasis and protecting nervous tissue against various pathological stimuli [18]. Under “resting” conditions, they in fact function as dynamic sensors that continuously “scan” their environment [19]. In the homeostatic state, they express specific surface markers such as transmembrane protein 119 (TMEM119) and purinergic receptor P2Y12 (reviewed in: Guerrrero and Sicotte, 2020, [20]). Depending on the insult, they exhibit different features, either protecting the tissue or exacerbating the injury. When activated under pathological conditions, microglial cells respond rapidly to neuronal distress changing their morphology from ramified to amoeboid. Quiescent state is characterized by small soma, multiple delicate processes, flattened nucleus, and small Golgi apparatus. While transforming to the active state, microglial cells enlarge the soma, retract processes, and overexpress immunomodulatory factors [21]. Two phenotypes of microglia have been classically distinguished: proinflammatory (M1) and anti-inflammatory (M2). This distinction is currently not obvious, as it has been suggested that microglial phenotypes are transient and demonstrate temporal and spatial profiles of transformation following an active response to the changes in the tissue microenvironment [22].

Microglia are implicated in multiple inflammatory and neurodegenerative diseases, including MS. These cells are a key player in the mechanisms underlying MS pathology exhibiting complex roles linked to the stage of the disease. They are critical for antigen presentation to T cells, development and exacerbation of inflammation, and subsequent synaptic loss. However, they can also modulate the inflammatory response and provide the protective functions by phagocytosis of tissue remnants and participation in the processes of tissue repair [23,24]. Moreover, phagocytosis of myelin debris in MS lesions, and expression of anti-inflammatory and protective factors by activated microglia, are essential processes to promote remyelination and enhance neuronal survival [25,26]. Protective factors released from microglia include neurotrophins such as nerve growth factor (NGF), brain-derived neurotrophic factor (BDNF), and basic fibroblast growth factor (bFGF) [21]. 

As immune competent cells of the CNS, microglia participate in immune response via interactions with other immune cells. There is a cross-talk between microglia and peripheral immune cells that are both present in MS lesions. Activated microglia, presenting MHCs antigens class I and II recognized by T cells, participate in the further recruitment of adaptive immune Th1 and Th17 cells into the CNS [27]. While interacting with cytotoxic T-lymphocyte-associated antigen 4 (CTLA4), microglia induce apoptosis of T cells, whereas the binding of microglia-derived molecules B7-1 and B7-2 to CD28 antigen stimulates the proliferation of T cells and release of cytokines [28].

In an early stage of MS, the initial pool of phagocytic cells in lesions is mainly comprised of microglia, as measured by their specific marker TMEM119 [29]. Increased expression of this marker was observed in MS patients [30]. As the disease progresses, peripheral macrophages are increasingly recruited [31]. Active demyelinating plaques in MS-diseased persons are occupied by phagocytic cells of both activated microglia and macrophages origin that contain products of myelin degradation and tissue debris, and highly expressed NADPH oxidase indicating oxidative stress-related damage [32]. Active demyelination is usually associated with a proinflammatory type of microglia, as indicated by the dominant expression of proinflammatory markers such as CD68 (involved in phagocytosis) and p22phox (involved in the production of reactive oxygen species), as well as CD86 and class II MHC antigens [29]. In the later stages of the disease, microglia/macrophages switches to an intermediate phenotype and co-expresses pro- and anti-inflammatory markers. Inactive lesions mainly consist of CD206-, CD163- and ferritin-positive microglia, indicating anti-inflammatory type [33]. In turn, the animal models of MS show that the presence of myelin debris in microglia is connected with a pro-regenerative phenotype expressing arginase-1, CD206, and insulin-like growth factor-1(IGF-1) necessary for remyelination [20,34].

Experimental evidence obtained using an EAE model of MS also confirms the significant impact of microglial pool of cells over the course of the disease. As reported, activation of microglia occurs in brains of EAE rats very early at a asymptomatic phase of the disease, as evidenced by highly increased immunoreactivity of microglia/macrophage-specific protein Iba-1 and morphological characteristics of microgliosis [35]. Regulation of microglia activity influences the outcome of the disease. The progression and the severity of neurological symptoms significantly declines after inhibition of macrophages/microglia at the developmental phase of the EAE, and the onset of the neurological deficits is delayed [36,37]. Targeting microglia activation by drugs such as minocycline, interferon-β, or fingolimod has been shown to have beneficial effects in reducing inflammation in both clinical and experimental studies of MS (reviewed in [38]). Finally, genetic factors found to be of importance for susceptibility to MS are more frequently related to microglia than other glial cells or neurons [39]. Over-expression of these risk genes highlights the significant contribution of microglia to the mechanisms underlying MS pathogenesis. 

### 2.3. Astroglia as Contributors to MS-Related Neuroinflammation

Astrocytes are the most abundant type of glial cells population of the CNS. These cells maintain the optimal microenvironment for neuronal function [40] and play a crucial role in a variety of processes related to the normal neuronal development, synaptogenesis [41], brain microcirculation, propagation of action potentials, or immunomodulation [42]. Astroglial processes surround neuronal synapses acting functionally as a tripartite synapse wherein they regulate ion and neurotransmitter homeostasis, support neurons metabolically, as well as control and regulate synaptic activity [43]. 

Astrocytes express a variety of, often specific, anion channels [44], hemichannels [40], and receptors, including ionotropic purinergic receptors [45]. It is well defined that the release of gliotransmitters (amino acids, ATP and peptides) into the extracellular space appears via Ca2+-dependent exocytosis [46]. It should also be emphasized that astrocytes act as an integrated system sensitive to signals derived from all cells that form the syncytium. 

A great amount of attention is paid to understanding of the mechanistic processes related to the astroglia dysfunction in response to immune attack, chronic neurodegenerative diseases, or brain injury. It is well accepted that, upon stimulation, morphologically and physiologically abnormal reactive astrocytes may interact with other cells in the CNS in a beneficial or negative way [47]. 

The characteristics of astrocytes and their perivascular location allow them to play a crucial role during lesion formation and to provide the access of peripheral immune cells into the CNS. In active lesions, astroglia acquire a hypertrophic morphology, as expressed by massive enlargement of the cell soma and reduced processes density that are most likely initiated by the failure of the astrocyte–oligodendrocyte network [9]. It is well established that, in the EAE experimental model of the disease, astrocytes become activated in the developing lesions before significant immune cell infiltration into the parenchyma, suggesting fundamental role of these cells in the lesion development [48]. Astrocyte activation associated with alterations in gene expression and cell hypertrophy was found to be followed by long-lasting scar formation and, in more advanced stages of the disease, with rearrangement of tissue structure. 

In the injured CNS, reactive astrocytes form a glial scar and are considered to be detrimental for axonal regeneration. Astrocyte activation appears via canonical signaling cascades, represented by NF-κB pathway that is crucial for neuroinflammation [49] and regulation of innate and adaptive immunity processes. Astrocytic NF-κB signaling is directly activated upon stimulation with the pro-inflammatory cytokines TNF-α and IL-1β, through TLR signaling and various other agents including myelin, mitogens, and free radicals [50]. The downstream NF-κB pathways in astrocytes are involved both in the initiation and exacerbation of inflammatory state in the CNS. A study using transgenic mouse model with astrocyte-specific disruption of NF-κB demonstrated a significant improvement of tissue damage and amelioration of clinical symptoms of EAE and spinal cord injury (SCI). 

One of the mechanisms by which astrocytes are activated in MS is a signal transducer and activator of transcription 3 (STAT3)-mediated pathway. It is well established that upregulation of STAT3 activity occurs after pathological stimulation followed by inflammation and injury of the CNS [51]. Pro- and anti-inflammatory pathways activate STAT3 signaling in astrocyte. Great examples are IFN-γ and IL-6 family cytokines that induce STAT3 phosphorylation by binding to the gp130 cell-surface receptor [52]. STAT3-mediated signaling in astrocytes was also characterized as a major player in inhibition of CNS inflammation in astrocyte-specific STAT3 knockouts. Herrmann et al. [53] showed that STAT3 deletion rendered astrocyte resistant to activation and astroglial scar formation, and resulted in active demyelination in the spinal cord lesions after SCI. Outlined changes were associated with the spread of inflammation and an increased volume of SCI-induced lesions. These findings clearly demonstrate that STAT3 signaling is a fundamental mediator of astrogliosis and provide additional evidence that scar-forming astrocytes inhibit the spread of inflammatory signals upon SCI [51]. Furthermore, EAE mice with astrocyte-specific deletion of the STAT3-activated gp130 receptor showed severe disease symptoms, as well as increased infiltration of reactive T-lymphocytes in the areas of demyelination [54].

It is worth noting that disruption of astrocyte–neuron integrity is a common hallmark of various human neurodegenerative diseases [55]. A recent study revealed that classically activated pro-inflammatory microglia secreting Il-1α, TNF, and C1q may induce reactivity of the astrocytes (known as a A1). The major sign of this astrocytic transformation is a lack of the neuroprotective features or gaining novel neurotoxic properties. An in vitro study using retinal ganglion cells co-cultured with A1 demonstrated the inhibition of the synapses development compared to those grown with control astrocytes. Furthermore, A1 astrocytes were found to secrete a soluble toxin that rapidly kills a subset of CNS neurons and mature oligodendrocytes via induction of the apoptosis [47]. Studies using in vivo approaches demonstrated that morphologically changed A1 astrocytes with numerous highly branched processes are mainly localized in the gray matter. Reactive astrogliosis is characterized by a range of functional changes that cause astrocytes unable to respond properly under pathological conditions. Interestingly, microglia-mediated activation of astrocytes and changes of astrocytic phenotype are abundant in almost all human neurodegenerative disorders, including MS. 

As outlined above, an increasing amount of evidence points towards the potential of reactive astrogliosis to play either primary or secondary roles in disease progression by disrupting normal glial functions or acquiring negative properties. It is also worth noting that astrocytes in pathological processes associated with MS can acquire both neurotoxic features related to the inflammatory signaling (regulation of leukocyte trafficking) and neuroprotective properties represented by the promotion of tissue repair. 

## 3. P2X7R-Mediated Signaling in Glial Cells 

### 3.1. Purinergic P2X7 Receptor—General Characteristic

Mammalian P2X7Rs are the members of the P2X purinoceptor family consisting of seven subtypes: P2X1, 2, 3, 4, 5, 6, and 7. These ligand-gated ion channels open in response to the extracellular agonist which is adenosine triphosphate (ATP). The P2X7R is a trimer composed of subunits, all of which share the common structure of two transmembrane domains, N- and C-terminal regions and large extracellular loop [56]. Structurally, P2X7R differs from other classes of the family in its long intracellular domain responsible for the pore formation. This specific receptor is activated by high concentrations of ATP in the millimolar range, whereas other members of P2XRs are stimulated by micromolar concentrations of ATP [57]. C-terminus of P2X7Rs, which is longer than in other P2XRs, has been identified as responsible for regulation of the receptor’s functions, including signaling pathways, cellular localization, protein–protein interactions, and post-translational modification [58,59].

Prolonged stimulation of P2X7Rs by ATP triggers formation of a non-selective pore, which allows the passage of molecules of up to 900 Da, Na^+^, and Ca^2^^+^ influx, and K^+^ efflux, resulting in changes in the ionic homeostasis of the cell [60]. In addition, the P2X7R may also mediate the large-scale release of intracellular ATP via its intrinsic pore or in connection with pannexin hemichannels thereby augmenting purinergic signaling and inflammation [61]. It is well known that P2X7-pannexin PANX1 pore complex critically determines spreading of depolarization followed by activation of neuroinflammatory machinery [62].

Inflammation-related events associated with P2X7R downstream signaling include the release of inflammatory mediators such as interleukin-1β and TNF-α. It is also becoming increasingly apparent that ATP-dependent signaling via the P2X7R plays a crucial role in astrocyte–neuron communications in a variety of pathological processes that occur in the central and peripheral nervous systems [4]. This prominent function of P2X7 receptors was strongly confirmed in a number of studies related to the neuronal degeneration, as well as behavioral or cognitive disorders.

The strong expression of P2X7R was identified predominantly in cells of haematopoietic linage such as monocytes, macrophages, mast cells, and microglia [63]. Within the CNS, this receptor is also expressed in neuronal processes [64], Müller cells [65], Schwann cells [66], oligodendrocyte precursor cells, and astrocytes [48]. The role of P2X7R-mediated signaling in glial cells, particularly in the context of stimulation and progression of the MS-associated pathology, will be discussed below in detail. 

### 3.2. P2X7R-Mediated Signaling in Microglia

Extracellular ATP is a potent signaling molecule, important in cell–cell communication in the CNS [67,68], which acts through an array of purinergic receptors, including the ATP-gated ion channels, the P2X7R. This specific type of purinergic receptors, widely expressed in the brain [69], has been shown to be substantially engaged in a variety of CNS pathologies, including MS [70]. However, the role of this receptor in the pathomechanisms and theclinical course of MS should be further clarified. 

The microglial expression of both quiescent and activated P2X7R in the nervous system has been reported [71,72]. As shown in primary hippocampal cultures, the overexpression of P2X7R is crucial for driving activation and proliferation of microglia [73]. It has also been confirmed that the activation of this type of receptor is strongly involved in the development and propagation of inflammatory reaction [74,75] by releasing a variety of proinflammatory cytokines such as interleukins: IL-1β, IL-18, IL-6, and tumor necrosis factor (TNF-α) [76]. 

P2X7R activation connected with pore formation [77] results in the outward blebbing of the microglial plasma membrane and the production of extracellular vesicles containing the proinflammatory cytokine, IL-1β, as well as the diffusion of ROS through the plasma membrane [78]. Recent data indicate that lysosomal exocytosis may be involved in the process of IL-1β release, as the lysosomal co-expression of IL-1β and P2X7R has been demonstrated [77]. Upon activation of the receptor, depolarization-induced K^+^ efflux and downstream signaling via a caspase 1-dependent mechanism leads to the activation of the NLRP3 inflammasome, a protein complex that participates in a proteolytic cleavage of an inactive form of interleukin 1β (pre-IL-1β) and the release of the active cytokine [79]. This primary event drives a self-propagating cycle via an autocrine mechanism and stimulates astrocytes via a paracrine action, thereby initiating the subsequent inflammatory cascade [21,80]. Stimulation of P2X7R is additionally linked to the activation of transcriptional factor NF-kB that up-regulates the IL-1β gene [81].

**Figure 1:**
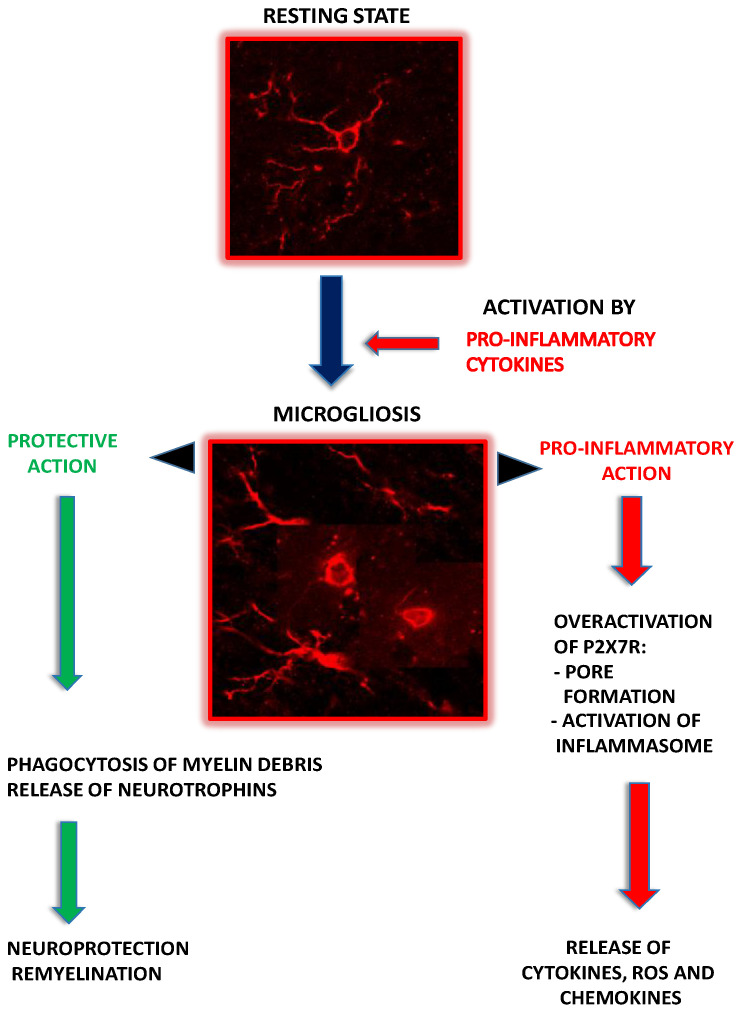
Schematic representation of the protective and negative roles of microglia and their P2XR in MS pathology. Activated microglial cells overexpress P2X7R whose overactivation, combined with the pore formation, mediates activation of inflammasome and the release of proinflammatory cytokines, chemokines, and reactive oxygen species (ROS) that exacerbate neuroinflammation. In parallel, protective functions of reactive microglia may be activated, such as phagocytosis of myelin debris, which subsequently triggers the release of neurotrophins and promotes remyelination (green arrows indicate protective effects, red arrows indicate negative effects). See text for details.

Microglia that express pore-forming P2X7R exhibit enhanced vesicular exocytosis and IL-1β release which then accelerates the trophic responses in microglia [77] and promotes activation and proliferation of these cells [82] with concomitant induction of neurotoxicity by stimulating the production of TNF-α and other toxic molecules such as IL-6 or reactive oxygen species [74]. P2X7R-dependent expression of ATP-induced pro-inflammatory tumor necrosis factor-α (TNF-α) is regulated in microglial cells by extracellular signal-regulated Kinase (ERK) and c-Jun N-terminal kinase (JNK) [74]. Further, strong evidence indicates that protein kinase δ (PKC δ) acts as an upstream regulator of ERK and JNKGGC [83]. It has also been shown that microglial P2X7R is important in regulating the expression and the release of not only proinflammatory cytokines, but also chemokines. ATP activates P2X7R in microglia with subsequent overexpression of mRNA and release of CXCL2 chemokine that facilitates neurotrophil infiltration. Calcineurin-dependent nuclear factor of activated T cells (NFAT) and protein kinase C (PKC)/MAP kinase (MAPK) are downstream pathways of P2X7R activation that are implicated in this chemokine release [84]. Moreover, via the intercellular communication, CXCL2 may potentiate the expression of other chemokines such as monocyte chemoattractant protein 1 (MCP-1, CCL2), 10 kDa interferon-induced protein (IP-10, CXCL10), and CCL5 in astrocytes [85]. The simplified scheme illustrating the contribution of microglia to the mechanisms underlying MS pathology is presented in Figure 1.

### 3.3. P2X7R-Mediated Signaling in Astroglia

Extracellular purine (ATP, ADP)-mediated signaling has been recognized as a dominant form of intercellular communication between astrocytes in the CNS, due to the ability of these cells to release purines to modulate neuronal activity and interact with other types of glia [86,87]. Moreover, purine-mediated astroglial signaling is disrupted in pathological states. It is well established that activation of astroglial P2X7Rs are closely associated with processes that initiate neuroinflammation and neuronal dysfunction. These events strongly depend on the pathological changes of astroglia that are related to the overproduction and uncontrolled release of cytokines, glutamate, and reactive oxygen species from astrocytes, all of which modulate astrocyte–neuron integrity and promote demyelination contributing to the neurodegenerative processes [88].

Numerous studies have established the capacity of astrocyte to maintain neuronal network homeostasis via a P2X7R-dependent purinergic signaling system. In the nervous system, ATP is released from neuronal axon terminals during the process of neurotransmission, as well as from astroglia. It is widely accepted that ATP is involved in synaptic transmission in many brain regions [89]. ATP can be stored and released either on its own or together with other neurotransmitters such as glutamate, GABA, noradrenaline or acetylcholine (ACh). Furthermore, activation of P2X7 receptors also triggers the release of gliotransmitters via exocytosis associated with the formation of P2X7-associated transmembrane pore channels (e.g., hemichannels, pannexins, volume sensitive anion channels). For instance, prolonged activation of P2X7Rs leads to a sustained glutamate release by hippocampal astrocytes [90]. High concentrations of ATP, acting through P2X7Rs, were also shown to significantly elevate production of endocannabinoid 2-arachidonoylglycerol in primary cultures of astrocytes [91]. Furthermore, stimulation of P2X7Rs in the same experimental condition has been found to act via different pathways including the release of TNF-α, stimulation of nitric oxide (NO) production, elevation of AKT, and p38MAPK/ERK1/ERK2 phosphorylation, or activation of transmembrane transport of NADH [92,93,94,95]. In addition, P2X7-mediated Ca^2+^ signaling was found to increase the production of lipid mediators of inflammatory cysteinyl leukotrienes [96]. Implication of P2X7 receptors in the processes related to the glutamate-glutamine pathway was observed in the RBA-2 astroglial cell line which was represented by a rapid decrease in glutamate uptake by the Na^+^-dependent transporter system and a decrease in the expression and activity of glutamine syntase. Furthermore, it is well established that P2X7 receptors are crucial in controlling expression of other purinoceptors and channels. For example, P2X7R stimulation increases expression of P2Y2 receptors and decreases expression of aquaporin-4 in primary cultured astrocytes [97].

It has been revealed that P2X7Rs expressed by primary cultured astrocytes may be activated in the absence of any exogenous stimuli, while knock-down of P2X7Rs decreases pore activity of the astrocytic receptor. Astrocytes incubated with an inhibitor of F-actin polymerization (CytD), an effective blocker of the phagocytosis, markedly reduced beads uptake by P2X7R in a manner dependent on actin filaments rearrangement [98]. Another study demonstrated that ATP stimulation of P2X7Rs causes a dissociation of MHC-IIa from its complex with P2X7Rs, resulting in decreased phagocytic activity [99]. These data suggest that basal activity of P2X7R expressed by resting astrocytes mainly regulates their engulfing features.

Constitutive activation of the astrocytic P2XRs is implicated in the clearance of the metabolic product of signaling molecule which is adenosine [100,101]. Proinflammatory stimulation with extracellular ATP via P2XRs at microlomar concentration significantly activates the release of purine nucleoside phosphorylase (PNP) by astrocytes in vitro. Interestingly, PNP release from glial cells partially occurred through the activation of the lysosomal pathway [102].

**Figure 2:**
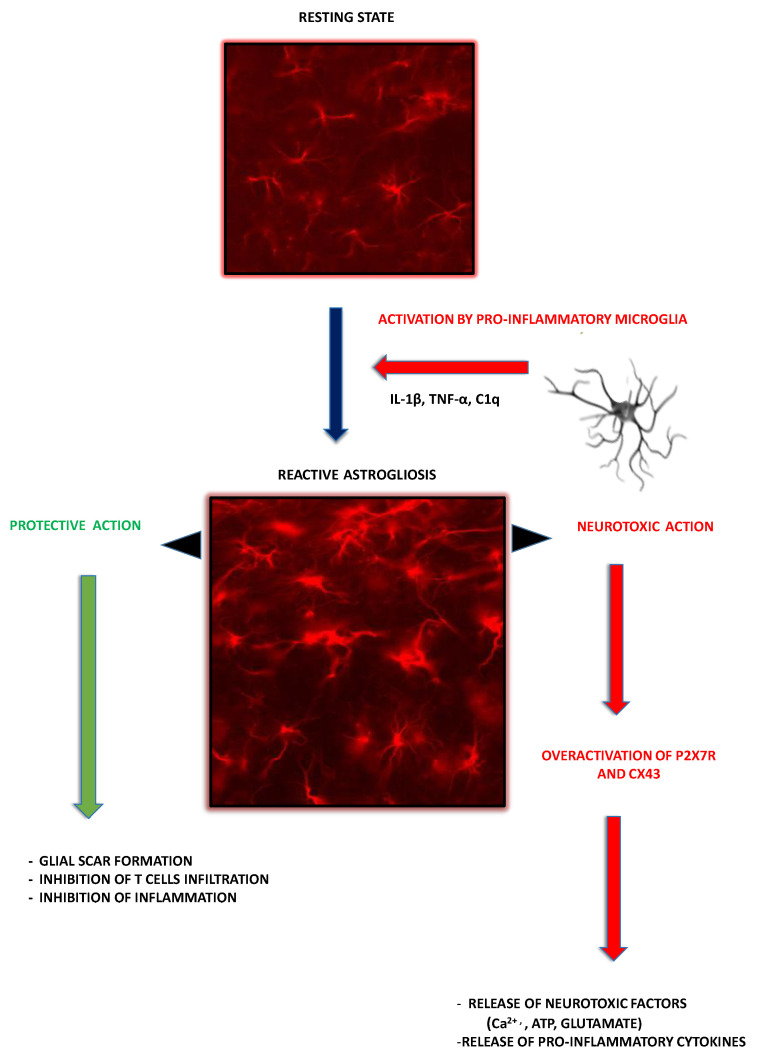
Schematic representation of the protective and negative roles of astroglia and their P2XR in MS pathology. Activated astrocytes overexpress P2X7R whose overactivation mediates efflux of neurotoxic factors such as extracellular ATP, calcium ions (Ca^2+^) and glutamate, as well as proinflammatory cytokines and chemokines that exacerbate neuroinflammation. In parallel, protective functions of reactive astroglia may be activated that are relevant to the processes of glial scar formation and remyelination. (green arrows indicate protective effects, red arrows indicate negative effects). See text for details.

The simplified scheme illustrating contribution of astroglia to the mechanisms underlying MS pathology is presented in Figure 2.

## 4. The Involvement of Glial P2X7R-Dependent Signaling into MS Pathology

### 4.1. Microglial P2X7R-Mediated Signaling in MS/EAE

In the context of MS/EAE pathology, P2XR has been first described to express in oligodendrocytes and myelin sheaths and to induce oligodendroglial cell death in vitro and in vivo. Functional P2X7R contributed to neuronal deficits in EAE animals and was also noticed in MS before lesion formation [103]. In post-mortem-collected MS tissue, increased expression of P2X7R in demyelinating plaques adjacent to blood vessels was shown within activated microglial cells/macrophages [104] where they released IL-1β via the induction of cyclooxygenase-2 and downstream pathogenic mediators [71]. Interestingly, in contrast to these results, in samples of frontal cortex obtained from secondary progressive form of MS, P2X7R expression was not detected either on resting or activated microglia [104]. 

Evidence from experimental studies using the EAE model of MS indicate that microglia are involved in inducing neuroinflammation via a P2X7R-dependent mechanism. P2X7R deficiency was shown to reduce the development of the disease in mice, inhibit inflammatory reaction, prevent ATP excitotoxicity in oligodendrocytes, and decline the axonal injury [103,105,106]. However, opposite results were also reported, describing exacerbation of the disease in P2X7R knockout mice [107]. Results of our studies showed that pharmacological blockade of P2X7R by its selective antagonist brilliant blue G (BBG) delays the onset of the disease and alleviates clinical symptoms in EAE rats. Moreover, we observed substantially inhibited activation and proliferation of microglia, as shown by decreased Iba-1 immunoreactivity and the morphological characteristics of microglial cells. Concomitantly observed lowered protein expression of proinflammatory cytokines, IL-1β, IL-6, and TNF-α, indicated inhibition of neuroinflammation. The P2X7R-dependent release of the proinflammatory cytokines was constantly decreased during the entire course of the disease after inhibition of the receptor with BBG [35]. Increased expression of P2X7R in microglial cells with concomitant up-regulation of several inflammatory genes associated with the activation of the NLRP3-inflammasome and the polarization of microglia to a pro-inflammatory phenotype was also observed in cuprizone model of demyelination [108]. Importantly, cuprizone-induced demyelination does not fully reflect the pattern of immune cell-related demyelination present in MS/EAE. However, P2X7R knockouts subjected to cuprizone toxicity showed attenuated micro- and astrogliosis, as well as the down-regulation of pro-inflammatory genes. The simplified summary illustrating the functional role of PX2R in different experimental models of MS pathology is presented in Table 1.

As mentioned, migration of activated peripheral immune cells, including monocytes, into the CNS is a critical step in the development of neuroinflammation in MS/EAE. Monocytes/macrophages are known to highly express P2X7R. The P2X7R-dependent release of CXCL2 chemokine was presented as an important factor in facilitating neutrophil infiltration in an experimental model of MS [110]. P2X7R protein expression was found to be diminished in monocytes in an acute phase of the disease, both in patients and EAE animals. Moreover, the protein levels of the receptor decreased in healthy monocytes subjected to pro-inflammatory stimuli in vitro. In MS tissue the receptor was lost on both CD14/CD68- or CD14/MHC-II-positive cells near the endothelium of the blood vessels [104]. The authors hypothesized that upregulation of P2X7R might be detrimental to monocytes, therefore secondary autocrine/paracrine down-regulation of P2X7R is triggered to support their survival and invasion into the CNS, thereby contributing to the induction and propagation of neuroinflammation.

### 4.2. Astroglial P2X7R 

In MS patients, P2X7R was found to be upregulated in the parenchymal astrocytes of frontal cortex from SP type of the disease [104]. Several pathological and morphological changes related to the reactive astrogliosis, such as hypertrophy, proliferation, and overlapping of cellular processes, resulting in the disruption of specific astrocytic domains, were revealed in immunochemical studies. In addition, P2X7R-positive astrocytes mediated glial scar formation in the white matter of chronic lesions. The same study revealed co-localization of the astrocytic P2X7R with the monocyte chemoattractant protein 1 (MCP-1), previously found to be responsible for the leukocyte recruitment during the progression of MS [111]. 

While modeling MS, it has been revealed that mice deficient in P2X7R function are more resistant to EAE than wild-type mice and exhibit reduction in the CNS inflammation-associated processes. Furthermore, within the CNS, astroglia-dependent axonal damage was present, while the opposite effect was observed in the P2X7R null mice. Furthermore, pharmacological inhibition of the receptor significantly abolished astrogliosis in rat EAE and reversed neurological symptoms [105]. These evidences strongly suggest the crucial role of astrocyte in MS pathology, which is associated with gaining new properties of the cells negatively affecting neuronal function. 

Studies from our laboratory focusing on the role of P2X7R-mediated signaling during the course of EAE revealed strong P2X7R expression within the frontal motor and somatosensory cortical brain regions, especially in the five and six layers of the cortex neighboring to the cingulum brain area. Interestingly, these changes were associated with the peak of neurological symptoms in immunized rats [109]. Another study using similar experimental conditions revealed the appearance of the astrogliosis in rat forebrains at an early stage of EAE. Overexpression of the specific astroglial markers occurred at fourth day post-immunization. Interestingly, at the same time, astrocyte overexpressed connexin 43 and P2X7R, while inhibition of the P2X7R signaling with BBG abolished activation of the astrocytes. Notably, administration of P2X7R antagonist partially reversed neurological symptoms developed during the disease progression. Given that astrocytes play an important role in the pathogenesis of CNS by releasing several potentially neurotoxic factors (e.g., ATP via purinergic system or glutamate), the dysfunction of activated astroglia suggests pathological involvement of glia cells in MS/EAE starting from an early stage of the disease [112].

As reported, overexpression of the astrocytic P2X7R in MS might be dependent on the disorder progression. Moreover, upregulation of the P2X7R activity seems to be associated not only with inflammatory reaction, but also with a variety of other processes related to the MS pathology, including the removal of glutamate excess, modulation of Ca^+^ and ATP efflux, glia scar formation, and lymphocyte homeostasis [113]. 

It is well accepted that activation of various astroglial receptors causes a transient increase in the intracellular pool of Ca^2+^ within the astroglia [114,115]. A consequence of the Ca^2+^ accumulation is a failure of astroglia to rapidly interact with neighboring cells in the CNS in physiological and pathological conditions [116]. Considerable evidence exists, supporting the role of Ca^2+^ signaling in astrocyte physiology. Synaptically released neurotransmitters mediate Ca^2+^ signaling in astrocytes, and action potentials along axons mediate the efflux of ATP and the intercellular propagation of astroglial Ca^2+^ signals. In turn, astrocytes amplify this initial signal by transmission of the extracellular Ca^2+^ wave to neighboring glia. Notably, propagation of these pathways was not observed in P2X7 knock-out mouse, providing the evidence that gliotransmitter-mediated signal propagation and amplification is strongly dependent on the P2X7 receptors [116]. The evidence also emerged that disruption of this pathway, negatively affecting the neuronal function, is present in a variety of pathological conditions such as neurodegenerative processes. 

The recent findings regarding the involvement of astroglia P2X7R in MS pathology revealed the rapid elevation of Ca^2+^ in primary culture of astrocytes upon addition of the isolated CNS-infiltrated immune cells (CNS IICs), predominantly represented by CD4+ T cells, recruited from the periphery to the CNS of EAE rats [117]. Interestingly, CNS IICs-stimulated Ca^2+^ elevation in astrocytes was markedly abolished by the specific block of P2X7 receptors, and was mimicked by the stimulation of this glial receptor with a low concentration of agonist. These results suggest that P2X7 receptor-dependent signaling primarily involves CNS IICs–astrocyte interaction. Furthermore, activation of P2X7 receptors appeared mainly in astroglia, while inhibition of the hemichannel-dependent ATP release in astrocytes declined Ca^2+^ accumulation, which was mediated after the addition of CNS IICs. Although this review focuses on the PX72R, it should be mentioned that other receptors abundantly expressed in glia cells represented by ionotropic P2X4, G protein-coupled P2Y_1_, and P2Y_2_ receptors are also involved in the regulation of intracellular Ca^2+^ concentration (more detail in [118]). Taken together, presented data suggest that rapid changes in the content of Ca^2+^ mediated by autoreactive immune cells involve astroglial purinergic signaling and lead to the long-lasting morphological and physiological changes of astroglia in EAE.

## 5. Conclusions

A growing body of evidence suggests heterogeneity of the processes related to MS pathology in which glial cells are strongly involved. The sensitivity of glial cells to different pathological stimuli and the potentiality to play dual role, positive or negative, point out their importance for neuronal functioning during the disease. The loss of glia protective functions, that is guarding and supporting of neuronal homeostasis, seems to be crucial for pathological processes running in MS-affected CNS.

Recent studies indicate the importance of purinoreceptor-mediated signaling in the glia–neuron cellular network. There is tempting evidence that suggests that activation of the P2X7R is commonly present during MS development and P2X7R-mediated purinergic signaling pathways, which drive and sustain neuroinflammation, significantly contributing to MS/EAE pathology. Taken together, numerous experimental and clinical observations indicate that both pools of glial cells that express P2X7R, microglia and astroglia, are involved in pathological mechanisms operating at early and progressive stages of the disease and should be considered as equally important in the pathogenesis of MS/EAE. Therefore, future studies will have to account for the potential role of purinergic P2X7R as a target for the promising therapeutic interventions in MS pathology.

## Figures and Tables

**Table 1:** Functional role of P2X7R during MS; results from experimental animal models.

P2X7 Targeting Approach	Model	Effect	Reference
Brilliant blue G (antagonist)	Acute EAE rats	Reduced onset of the disease; reduced astrogliosis and microglia proliferation	[35] [109]
Brilliant blue G (antagonist)	Chronic EAE mice	Reduced demyelination; ameliorated neurological abnormalities	[103]
Oxidized ATP (oxATP antagonist)	Chronic EAE mice	Inhibition of clinical symptoms and demyelination; reduce antigen T cell	[106]
Cuprizone (demyelination inducer)	P2X7 null mice	Inhibition of astrogliosis and microglia activation	[108]

## Data Availability

Not applicable.
